# Effects of imidapril treatment on aquaporin-2 expression in the kidneys and excretion in the urine of hypertensive rats

**DOI:** 10.3892/etm.2013.1014

**Published:** 2013-03-15

**Authors:** WEI ZHAO, AI-GUO XU, JING WU, JING GUO, QIN-FU XU, DAN-DAN LI, YU-MIAO ZHAO

**Affiliations:** Department of Critical Care Medicine, The First Affiliated Hospital of Zhengzhou University, Zhengzhou, Henan 450052, P.R. China

**Keywords:** imidaprilat, hypertensive rats, aquaporin-2, arginine vasopressin

## Abstract

Renal aquaporin-2 (AQP2) is critical for maintaining water balance and is associated with hypertension. Anti-hypertensive drugs, including imidapril, improve kidney function; however, it remains unclear whether these effects are mediated through the regulation of AQP2. In this study, the effects of imidapril on AQP2 expression in the kidneys and excretion in urine were assessed in hypertensive rats. Hypertension was induced in 24 rats, which were randomized into a control group, treated with water only, and an imidapril treatment group (n=12 per group). Blood and urine samples were collected from all rats to determine blood pressure (BP), serum Na^+^, urine volume and urine osmolality after 8 weeks of treatment. Molecular and immunological techniques were used to measure the expression of AQP2 in the kidneys. Urine AQP2 concentration was detected by indirect enzyme-linked immunosorbent assay (ELISA). The concentration of plasma arginine vasopressin (AVP), a regulator of AQP2 was detected by radioimmunoassay (RIA). Hypertensive rats treated with imidapril exhibited reduced BP and 24-h urine osmolality, with a concomitant increase in 24-h urine volume, compared with control hypertensive rats (P<0.05). Additionally, the expression of *Aqp2* mRNA, detected by RT-PCR, and AQP2 protein, detected by immunohistochemistry and western blotting, in renal tissue significantly decreased (P<0.05). Finally, urine AQP2 concentration increased while plasma AVP concentration decreased following imidapril treatment (P<0.05). These findings indicate that imidapril reduces the expression level of AQP2 in renal tissue and accelerates its excretion.

## Introduction

Aquaporin-2 (AQP2), a member of the AQP family, plays a vital role in regulating the water balance in the body. This protein is primarily observed in the collecting duct of the kidneys (1). A significantly increased expression of *Aqp2* mRNA has been observed in the kidneys of rats with spontaneous hypertension (2,3). Similarly, clinical observations indicate that the urine AQP2 concentration is increased in hypertensive patients with low renin levels (4). These findings highlight a correlation between AQP2 expression and the occurrence of hypertension.

AQP2 is regulated by the neurohypophysial hormone arginine vasopressin (AVP or AVP-V2) through the AVP receptor on AQP2 (3). The AVP receptor is regulated, in part, by angiotensin II (AngII), a peptide hormone that induces vasoconstriction (5). AngII is formed by the conversion of angiotensin I (AngI) by angiotensin-converting enzyme (ACE) (6). ACE inhibitors, including imidapril, block the conversion of AngI to AngII (7). A previous study of another ACE inhibitor, enalapril, indicated that treatment of hypertensive rats with enalapril reduces the overexpression of AQP2 and AVP-V2 in the kidneys (8). However, it is not known whether imidapril is able to regulate the expression or secretion of AQP2.

In the current study, changes in AQP2 expression in the kidneys, AQP2 concentration in the urine and plasma AVP concentration in hypertensive rats were detected by molecular, immunological and biochemical techniques prior to and following the administration of imidapril. These analyses are likely to advance our understanding of the regulation of AQP2 by ACE inhibitors in hypertension.

## Materials and methods

### 

#### Rats

Pathogen-free male Wistar rats (body weight, 120–140 g) were purchased from Vital River Lab Animal Technology (Beijing, China). Rats were housed three or four per cage and maintained at constant temperature (22±2°C) and constant humidity (55±5%) with a 12-h light/dark cycle. Food and water were available *ad libitum.* To induce hypertension, rats were gavaged at 40 mg/kg/day with N-nitro-L-nitro-arginine-methyl-ester (L-NAME; Sigma, St. Louis, MO, USA), a nitric oxide synthase inhibitor. On week 3, caudal arterial blood pressure (BP) and heart rate were determined with an RBP-I-type rat BP and heart rate machine (Clinical Research Institute, China-Japan Friendship Hospital, Beijing, China). Rats with systolic BP >18.67 kPa (140 mmHg; 24 rats) were then randomized to either the control group or the imidapril treatment group (n=12 per group). Following measurements of BP and body mass, rats in the treatment group received an intragastric injection of 2.5 mg/kg/day imidapril (Tianjin Tanabe Seiyaku Co., Ltd., China) at 09:00 daily for 8 weeks. Control rats received an equal volume of water in the same manner. BP and body mass measurements were performed once a month.

#### Specimens

Rats were placed in metabolic cages prior to decapitation to collect 24-h urine output. Freshly collected urine was centrifuged at low temperature at 2,000 rpm for 5–10 min. The supernatant was collected and centrifuged at low temperature at 2,000 rpm for 75 min using an ultrafiltration tube (centriplus-10; Millipore, Billerica, MA, USA) to concentrate the urine to <1 ml. Concentrated urine was stored at −70°C for future use. Rats were anesthetized intraperitoneally with 10% chloral hydrate at 3 ml/kg and decapitated following blood collection. Kidneys were rapidly removed and washed with phosphate-buffered saline (PBS). The renal medulla was separated according to gross anatomy and placed in a tube containing liquid nitrogen. Blood samples (1 ml) were collected in 0.13 mM disodium ethylenediamine tetraacetate (EDTA-2Na) solution (20 ml/l whole blood) and centrifuged at 4°C at 3,000 rpm for 10 min. Plasma was collected and stored at −70°C for detection of AVP concentration. The urine osmolality was detected by the freezing point inhibition method and serum Na^+^ concentration.

#### Plasma AVP concentration

The plasma AVP concentration was measured by radioimmunoassay using a plasma AVP radioimmunoassay kit (DSL Biological Products, Webster, TX, USA) according to manufacturer’s instructions. The FM-2000γ immune counter (Xi’an Kaipu Electrical, China) was used to detect radiolabeling.

### AQP2 expression in the kidney

#### Immunohistochemistry

Tissues near the renal medulla were fixed in neutral formalin for 24 h, then treated using conventional histological methods for paraffin embedding. Paraffin-embedded tissues were sectioned at 4 *μ*m with a paraffin slicer (Leica RM2016, Wetzlar, Germany) and collected on glass slides. Sections were dewaxed with xylenes and rehydrated with an alcohol gradient. Antigen repair was performed with citric acid solution and microwave heating. Room-temperature slides were treated with 3% hydrogen peroxide to block activity of endogenous peroxidase. Sections were then covered with non-specific serum in a humidified box and incubated at room temperature. The primary antibody against AQP2 (rabbit anti-rat/mouse IgG; Calbiochem, San Diego, CA, USA) was applied to sections, which were then incubated in a humidified box at 4°C overnight. For the negative control, goat serum was used in place of the primary antibody. After washing three times with PBS, 50 *μ*l biotin-labeled secondary antibody (Santa Cruz Biotechnology Inc., Santa Cruz, CA, USA) were added and the sections were incubated at room temperature. After washing three times with PBS, 50 *μ*l ready-to-use streptavidin-horseradish peroxidase (HRP, SP kit; Maixin Inc., Fuzhou, China) was added to the sections, which were then incubated at 37°C for 30 min. 3,3′-Diaminobenzidine (DAB) substrate (Maixin Inc.) was applied to develop color. Staining results were observed under a light microscope (Olympus, Tokyo, Japan) before termination of the reaction. Sections were then counterstained with hematoxylin, dehydrated and mounted for visualization. Positive staining appears as brownish-yellow puncta in the cell membranes and cytoplasm of the kidney specimens. Image-Pro Plus 6.0 image analysis software (Media Cybernetics Inc., Silver Spring, MD, USA) was used to detect staining. Five visual fields were randomly selected to determine the percentage of positively-stained cells.

#### RT-PCR

Total RNA was extracted from 100 mg tissue near the renal medulla using a TRIzol RNA extraction kit (Takara Biotech Co., Ltd., Dalian, China). The A260/A280 ratio of the total RNA (752C spectrophotometer Shanghai Spectrum Instruments Co., Ltd., Shanghai, China) from collected samples was calculated to be 118–210. Total RNA (2 *μ*g) was used for reverse transcription to synthesize cDNA using M-MuLV (MBI Fermentas, Burlington, Canada) as reverse transcriptase in a 20-*μ*l reaction. cDNA (2 *μ*l) was amplified by the GeneAmp PCR system with 18S rRNA (forward: 5′-CGACGGACCCATTCGAACGTCT-3′ and reverse: 5′-GCTATTGGAGCTGGAATTACCG-3′) and *Aqp2* (forward: 5′-CATGTCTCCTTCCTTCGAGC-3′ and reverse: 5′-TTGTGGAGAGCATTGACAGC-3′) primers and Taq polymerase (Bio-Asia Diagnostics Co., Ltd., Shanghai, China). Based on the published sequence of *Aqp2* (GenBank Accession No. NM_000486), the primers were designed and synthesized by Takara Biotech Co., Ltd. The following reaction conditions were used: initial incubation for 5 min at 95°C, followed by 30 cycles of 30 sec at 94°C, 30 sec at 60°C, 60 sec at 72°C and 7 min at 72°C. PCR products were separated in ethidium bromide 2% sepharose gels and visualized with a gel imaging system. The expected sizes of the amplification products were 131 bp for *Aqp2* and 312 bp for 18S rRNA. 18S rRNA was used as the internal standard to perform semi-quantitative analysis for PCR products, for which relative absorbance was measured using image analysis software.

#### Western blotting

Kidney tissues were homogenized with histone solution using a Polytron high-speed homogenizer. Proteins were denatured by boiling samples for 3 min. Proteins were separated via 12% sodium dodecyl sulfate (SDS)-polyacrylamide gel electrophoresis prior to transfer to a nitrocellulose membrane. Proteins on nitrocellulose membranes were blocked with 5% skimmed milk at 37°C for 2 h, then AQP2 primary antibody (rabbit anti-rat/mouse IgG) was added to the membrane for incubation at 4°C overnight. Membranes were washed with Tris-buffered saline with Tween-20 (TBST) at 37°C three times for 10 min each. Biotin-labeled secondary antibody (Santa Cruz Biotechnology Inc.) was added prior to incubation at 37°C for 40 min. Following three more washes with TBST, the staining color was developed by the addition of an enhanced chemiluminescence (ECL) reagent for 3–5 min. A gel imaging system was used to visualize and compare the protein bands. Expression was normalized against β-actin, which was used as an internal control.

#### AQP2 concentration in urine

The AQP2 concentration in the urine was determined by enzyme-linked immunosorbent assay (ELISA) according to previously published methods (9,10). Briefly, the square matrix titration method was used to detect the optimum working concentration of antibodies; the primary antibody (rabbit anti-AQP2 purified polyclonal antibody) was optimized at 2 mg/l and the secondary antibody (HRP goat anti-rabbit IgG; New England Biolabs, Beijing, China) was optimized at 1:1000. The AQP2 positive control, polypeptide standard preparation coupled with bovine serum albumin (BSA; Alpha Diagnostic, San Antonio, TX, USA), was diluted with 0.05% SDS-PBS at a 1:1 ratio. Then, 100 *μ*l diluted urine samples and 100 *μ*l standard preparation were added to a 96-well plate. Negative control wells contained 100 *μ*l 0.05% SDS. Plates were pre-coated at 37°C for 30 min, then incubated at 4°C overnight. Each well was washed with 0.05% PBS with Tween-20 (PBST) and incubated with 100 *μ*l blocking solution (3% BSA-PBS) at 37°C for 45 min. Next, 0.25% BSA-PBS was added to each well (diluted to 100 *μ*l). The primary antibody against AQP2 (2 mg/l) was added to the wells and plates were incubated at 37°C for 2 h. Following four 1-min washes with PBST, the secondary antibody diluted with 0.25% BSA-PBS was incubated in the wells at 37°C for 90 min. Following another four washes with PBST, 100 *μ*l fresh 0.01% TMB substrate buffer was added to each well for incubation at 37°C for 15 min. Finally, 2 *μ*M H_2_SO_4_ was added to each well to terminate the reaction. Absorbance was measured at 450 nm on a microplate reader (ELX800 enzyme-linked immunosorbent detector; Dio-Tek Instruments Inc., Winooski, VT, USA). The standard curve was determined using the absorbance of the standard solution.

### Statistical analysis

SPSS 17.0 statistical software (SPSS Inc., Chicago, IL, USA) was used for statistical analyses. Measurement data are expressed as the mean ± standard deviation. The independent-sample t-test was used to analyze and compare differences in the intergroup indices. α=0.05 and P<0.05 were considered to indicate a statistically significant difference.

## Results

### 

#### Imidapril affects BP, urine output and urine osmolality in hypertensive rats

All experimental rats were induced to exhibit hypertension, then randomly assigned to a control group (water) or treatment group (imidapril). Following treatment, the rats were assessed for changes in BP and kidney function (Table I). Compared with rats in the control group, rats treated with imidapril exhibited decreased systolic BP, increased 24-h urine output and decreased urine osmolality (P<0.05). However, no significant difference was observed in Na^+^ level between the two groups.

#### Effects of imidapril on AQP2 in the kidneys of hypertensive rats

**Figure 1:**
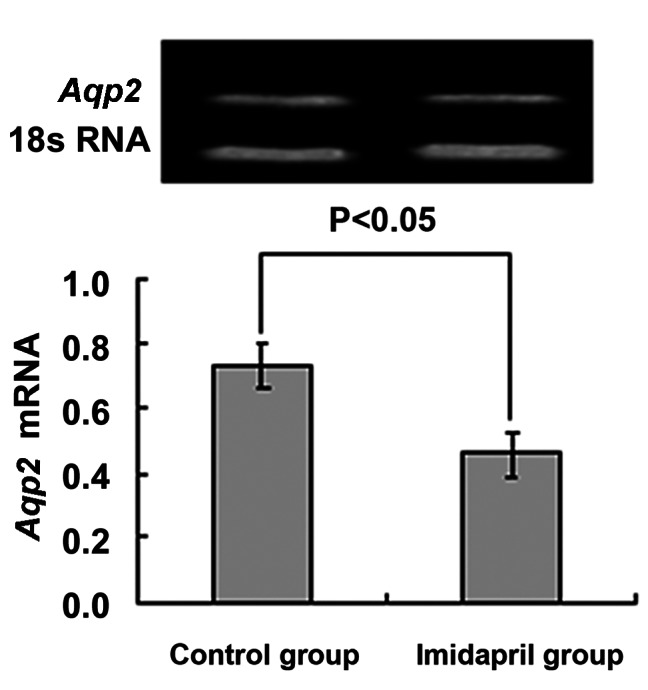
Relative mRNA expression of aquaporin-2 (AQP2) in control and imidapril-treated hypertensive rat kidneys. RT-PCR for 18S rRNA was included as an internal control. The expression of *Aqp2* was normalized against 18S rRNA expression.

To determine whether the modulating effects of imidapril on kidney function in hypertensive rats involved changes in AQP2 expression, we used molecular and immunological techniques to assess the expression of *Aqp2* mRNA and the protein product. Semi-quantitative mRNA expression was determined by RT-PCR in hypertensive rats in the control and imidapril-treated groups and was normalized against 18S rRNA expression. Compared with the control group (0.73±0.07), relative *Aqp2* expression in imidapril-treated rat kidneys was significantly lower (0.46±0.07, t=9.263, P=0.001; Fig. 1).

**Figure 2:**
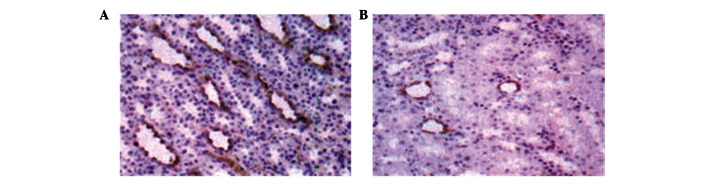
Immunohistochemistry for aquaporin-2 (AQP2) demonstrated the expression of AQP2 primarily in cells surrounding the collecting tubes in renal tissues of hypertensive rats. Note the reduced staining intensity in (B) imidapril-treated rat kidneys compared with (A) control rat kidneys.

**Figure 3:**
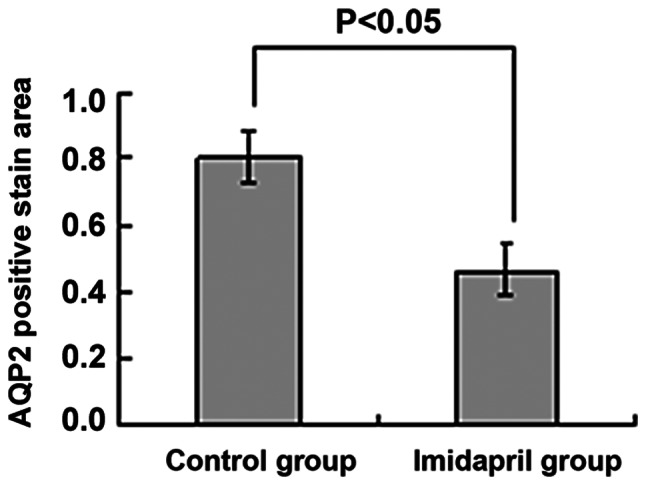
Positive staining area of aquaporin-2 (AQP2) in the renal tissue of control and imidapril-treated hypertensive rats.

Immunohistochemistry against AQP2 was performed on sections from hypertensive rat kidneys. As expected, AQP2 was detected predominantly in cells surrounding the collecting tube in tissues near the renal medulla (Fig. 2). However, AQP2 staining in the kidneys of imidapril-treated rats was significantly lighter (Fig. 2B) compared with that of rats in the control group. Additionally, the positively stained area (0.46±0.07) of kidney sections from rats in the imidapril group was significantly smaller compared with that of the control group (0.80±0.08, t=11.154, P=0.001; Fig. 3).

**Figure 4:**
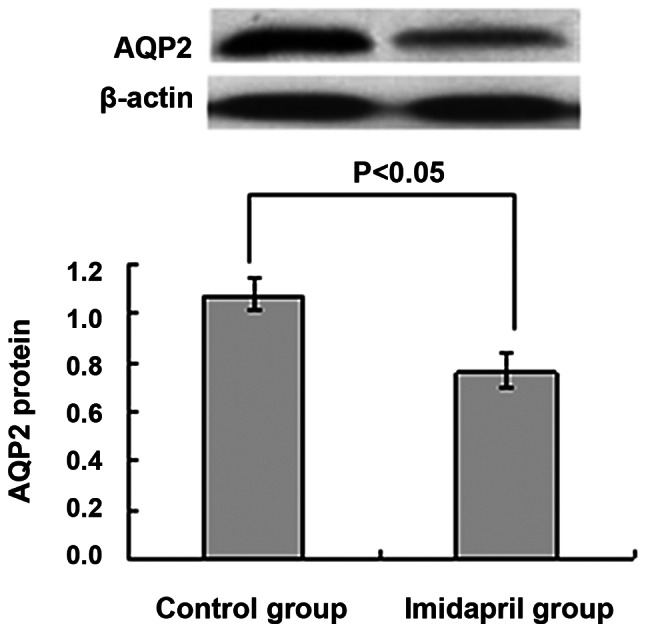
Western blot analysis of relative aquaporin-2 (AQP2) expression in the renal tissue of control and imidapril-treated hypertensive rats. AQP2 expression was normalized against the expression of β-actin.

Given the reduced staining intensity observed by immunohistochemistry, we sought to quantify AQP2 expression in rat kidneys using western blot analysis. AQP2 expression was normalized against β-actin expression. Relative AQP2 expression in the kidneys of imidapril-treated rats (0.76±0.06) was significantly lower compared with that of rats in the control group (t=10.371, P=0.001; Fig. 4).

#### Imidapril affects plasma AVP and urine AQP2 concentration in hypertensive rats

Imidapril treatment significantly altered the concentration of AVP in the plasma and of AQP2 in the urine of hypertensive rats (Table II). Compared with the control group, the plasma AVP concentration of imidapril-treated rats was significantly reduced; by contrast, the urine AQP2 concentration was significantly increased following imidapril treatment (P<0.05).

## Discussion

AQP2 is the key protein regulating the water permeability of the renal collecting duct; therefore, it is critical in maintaining the renal water balance (10,11). AQP2 operates through short- and long-term regulatory mechanisms (12–14). AQP2 is also the only AVP-dependent AQP. Elevated AVP content in plasma promotes AQP2 expression in the epithelium of the renal collecting duct, opening the water channel and increasing water reabsorption, which results in urine concentration and increased infiltration capacity (15).

The renin-angiotensin-aldosterone system (RAAS) is an endocrine pathway that regulates water and electrolyte balance, blood volume and BP through a number of hormones and enzymes (16,17). Renin is a proteolytic enzyme that is synthesized and secreted by the juxtaglomerular cells (18) and promotes the conversion of plasma pro-angiotensin to AngI (19). The conversion of AngI by ACE produces AngII, a BP-boosting protein that promotes the vasoconstriction of small arteries and indirectly increases BP (20). AngII also stimulates the adrenal zona to produce greater quantities of aldosterone, promotes the absorption of sodium and chloride ions by distal tubules, increases blood volume and leads to increased BP.

Imidapril, a new type of highly selective ACE inhibitor, when administered orally, becomes the active metabolite, imidaprilat, through liver de-esterification (20,21). Imidaprilat inhibits the activity of ACE and prevents the conversion of AngI to AngII. This causes peripheral vasodilatation and reduces vascular resistance, thus producing an antihypertensive effect (7). Additionally, imidapril reduces aldosterone secretion, increases Na^+^ discharge and simultaneously reduces glomerular perfusion pressure and increases renal blood flow, thus increasing urine volume (22). Therefore, it is not surprising that we identified that imidapril treatment significantly reduces BP and urine osmolality while also increasing 24-h urine output in hypertensive rats.

By investigating the changes induced by imidapril treatment, we sought to determine whether treatment affects the expression of AQP2. mRNA and protein levels were reduced in imidapril-treated hypertensive rats compared with control hypertensive rats. Furthermore, urine AQP2 concentrations were significantly increased following imidapril treatment. These findings indicate that imidapril downregulates AQP2 expression in renal tissues and increases AQP2 urine excretion. The inhibitory effect of imidapril on AQP2 expression may involve imidapril preventing AngII generation and subsequently reducing the stimulatory effect of AngII on the expression of AVP-V2 receptor mRNA, thus indirectly inhibiting the expression of AQP2. We also identified that plasma AVP concentrations significantly decreased. Since AQP2 is mainly located in the cytoplasm of cells and the membrane of tubules of the renal collecting duct and a certain amount of AQP2 protein enters the tubules and is washed away by urine, the AQP2 concentration in urine is correlated with the effects of AVP in plasma and AQP2 expression in the kidney.

## Figures and Tables

**Table I:** Comparison of systolic pressure, Na^+^ concentration, 24-h urine output and urine osmotic pressure in hypertensive rats left untreated or treated with imidapril.

Treatment group	n	Systolic pressure (mmHg)	Na^+^ (mmol/l)	24-h urine volume (ml)	Osmotic pressure (mOsm/kg H_2_O)
Control	8	155.1±17.6	146.3±6.8	11.3±2.1	1818.6±118.6
Imidapril	8	132.3±20.1	151.3±6.3	17.0±2.2	1311.8±77.4
t-value		2.965	1.862	6.653	12.393
P-value		0.007	0.076	0.001	0.001

**Table II:** Comparison of plasma AVP and AQP2 concentrations in hypertensive rats left untreated or treated with imidapril.

Treatment group	n	AVP (ng/l)	AQP2 (*μ*g/l)
Control	8	81.9±12.0	12.2±1.3
Imidapril	8	50.2±8.6	19.9±3.3
t-value		7.439	7.466
P-value		0.001	0.001

AVP, arginine vasopressin; AQP2, aquaporin-2.
